# Cost-effectiveness of population-based screening for colorectal cancer: a comparison of guaiac-based faecal occult blood testing, faecal immunochemical testing and flexible sigmoidoscopy

**DOI:** 10.1038/bjc.2011.580

**Published:** 2012-02-16

**Authors:** L Sharp, L Tilson, S Whyte, A O'Ceilleachair, C Walsh, C Usher, P Tappenden, J Chilcott, A Staines, M Barry, H Comber

**Affiliations:** 1 National Cancer Registry Ireland, Cork Airport Business Park, Building 6800, Kinsale Road, Cork, Ireland; 2 National Centre for Pharmacoeconomics, St James's Hospital, Dublin, Ireland; 3 School for Health and Related Research, University of Sheffield, Sheffield, England; 4 Department of Statistics, Trinity College Dublin, Dublin, Ireland; 5 School of Nursing, Dublin City University, Dublin, Ireland

**Keywords:** colorectal cancer, mass screening, cost-effectiveness, FOBT, faecal immunochemical test, flexible sigmoidoscopy

## Abstract

**Background:**

Several colorectal cancer-screening tests are available, but it is uncertain which provides the best balance of risks and benefits within a screening programme. We evaluated cost-effectiveness of a population-based screening programme in Ireland based on (i) biennial guaiac-based faecal occult blood testing (gFOBT) at ages 55–74, with reflex faecal immunochemical testing (FIT); (ii) biennial FIT at ages 55–74; and (iii) once-only flexible sigmoidoscopy (FSIG) at age 60.

**Methods:**

A state-transition model was used to estimate costs and outcomes for each screening scenario *vs* no screening. A third party payer perspective was adopted. Probabilistic sensitivity analyses were undertaken.

**Results:**

All scenarios would be considered highly cost-effective compared with no screening. The lowest incremental cost-effectiveness ratio (ICER *vs* no screening €589 per quality-adjusted life-year (QALY) gained) was found for FSIG, followed by FIT (€1696) and gFOBT (€4428); gFOBT was dominated. Compared with FSIG, FIT was associated with greater gains in QALYs and reductions in lifetime cancer incidence and mortality, but was more costly, required considerably more colonoscopies and resulted in more complications. Results were robust to variations in parameter estimates.

**Conclusion:**

Population-based screening based on FIT is expected to result in greater health gains than a policy of gFOBT (with reflex FIT) or once-only FSIG, but would require significantly more colonoscopy resources and result in more individuals experiencing adverse effects. Weighing these advantages and disadvantages presents a considerable challenge to policy makers.

In 2008, an estimated 1.2 million new cases of colorectal cancer were diagnosed worldwide and 600 000 people died from the disease (Ferlay *et al*, 2010). It is the third most common cancer in men and second most common in women. In light of this burden, various international organisations have strongly recommended implementation of colorectal cancer screening (Council of the European Union, 2003; US Preventive Services Task Force, 2008).

Several screening tests are available which, by detecting benign adenomas or early cancers, might reduce colorectal cancer mortality and, potentially, incidence in the population. Until recently, the only test for which there was robust evidence of efficacy from randomised controlled trials (RCTs) was the guaiac-based faecal occult blood test (gFOBT). Repeated screening with gFOBT results in a 16% mortality reduction (Hewitson *et al*, 2007), evidence which underpinned the decision to base the UK colorectal cancer-screening programmes on gFOBT. However, gFOBTs have several weaknesses, including limited sensitivity even when used biennially (Burch *et al*, 2007; van Dam *et al*, 2010). In addition, substantial proportions of screened individuals need to complete a second (reflex) test before a decision is taken on referral for diagnostic investigation by colonoscopy. Recent years have seen the development of faecal immunochemical tests (FIT), which are specific for bleeding of colorectal origin and do not require individuals to undergo dietary restrictions (van Dam *et al*, 2010). Although evidence is lacking on whether FIT-based screening is effective in reducing mortality, RCTs show that these tests have a higher neoplasia detection rate and positive predictive value than gFOBTs (van Rossum *et al*, 2008; Hol *et al*, 2010; Levi *et al*, 2011). In addition, some studies show a higher uptake with FIT (Cole and Young, 2001; Deutekom *et al*, 2010; Hol *et al*, 2010). On this basis, FIT is now considered an acceptable screening option by various bodies (Levin *et al*, 2008). However, compared with gFOBT, FIT kits are generally more expensive and (older) qualitative FITs have a lower analytical detection limit, resulting in a high colonoscopy referral rate (Fraser *et al*, 2007), both key considerations for publicly funded screening programmes. Newer quantitative FITs allow the cut-off to define a ‘positive’ result to be varied (Hol *et al*, 2009), which provides a potentially attractive way to control colonoscopy requirements. A different way of managing the challenge of colonoscopy referrals has been adopted in the screening programme in Scotland, where FIT is used for reflex testing following a positive gFOBT. This has reduced the proportion of screened individuals referred for colonoscopy compared with using second-line gFOBT (Fraser *et al*, 2006, 2007). However, the potential impact of gFOBT with reflex FIT on cancer incidence and mortality does not appear to have been evaluated.

An alternative approach to screening is to use an endoscopy-based test. This option is firmly back on the agenda following publication of results from three RCTs (Hoff *et al*, 2009; Atkin *et al*, 2010; Segnan *et al*, 2011). In two trials, a single examination between age 55 and 64 was associated with significant reductions in colorectal cancer incidence and/or mortality (Atkin *et al*, 2010; Segnan *et al*, 2011) whereas in the other, although incidence was not reduced in those randomised to screening, a non-significant decrease in mortality was observed (Hoff *et al*, 2009).

Cost-effectiveness analysis provides a methodology for comparing the costs and benefits of alternative healthcare interventions. Evaluations of cost-effectiveness of colorectal cancer screening have been conducted in several countries (HIQA, 2009; Landsdorp-Vogelaar *et al*, 2010, and references therein). Most suggest that screening – by one of a range of different tests and strategies – is likely to be considered cost-effective compared with no screening. However, uncertainty remains. None of the screening tests appear optimal across all settings (Lansddorp-Vogelaar *et al*, 2010) and it is unclear which of the current options are likely to provide the best balance of costs and benefits. In particular, the evidence based around FIT is limited (Berchi *et al*, 2010; Telford *et al*, 2010; Lejeune *et al*, 2010; Hassan *et al*, 2011; van Rossum *et al*, 2011), and it does not appear to have been evaluated alongside once-only flexible sigmoidoscopy (FSIG). The aim of this study was to estimate the incremental cost-effectiveness of a population-based colorectal cancer-screening programme using primary FIT, gFOBT with reflex FIT testing, or once-only FSIG compared with no screening.

## Materials and methods

### Setting and screening scenarios

The study setting was Ireland. Ireland has a mixed public-private health care system, but population-based cancer screening programmes are provided free at the point of delivery. Three primary screening scenarios were evaluated: (1) biennial gFOBT, with reflex FIT, in those aged 55–74 years; (2) biennial FIT in those aged 55–74 years; and (3) once-only FSIG at age 60. In secondary analyses, five age-variant scenarios were considered: (1) biennial gFOBT, with reflex FIT, at 55–64 years; (2) biennial gFOBT, with reflex FIT, at 65–74 years; (3) biennial FIT at 55–64 years; (4) biennial FIT at 65–74 years; and (5) once-only FSIG at age 55. This evaluation was conducted to inform health policy and decision-making in Ireland, and the screening scenarios were determined largely by an Expert Group established to oversee the evaluation. It was assumed that investigation of positive screening tests would be by colonoscopy, with CT colonography in those unfit for colonoscopy, or in whom colonoscopy was incomplete. Follow-up of individuals who had adenomas detected and removed was assumed to follow existing guidelines (Atkin and Saunders, 2002). Briefly, those who had low-risk adenomas removed would return to routine screening and those who had intermediate or high-risk adenomas removed would enter ongoing colposcopic surveillance, conducted annually for those with high-risk findings and every 3 years for those with intermediate-risk findings. Individuals would exit surveillance after two clear tests 3 years apart.

### Economic model structure

The economic model was a state transition (Markov process) model with three interlinked components relating to the: (1) natural history of colorectal neoplasia; (2) impact of screening and subsequent adenoma surveillance; and (3) impact of mortality.

**Figure 1:**
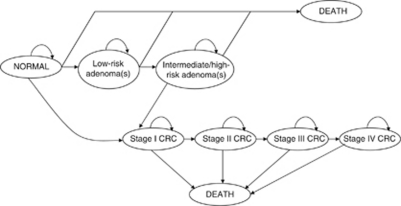
Simplified schematic of natural history model states and transitions. Low-risk adenoma: <10 mm; intermediate/high-risk adenomas: ⩾10 mm; Abbreviation: CRC=colorectal cancer.

The natural history model simulated the experience of a cohort of individuals over their lifetime through health states relating to the progression from normal colorectal epithelium, through the adenoma-carcinoma sequence, to death (Figure 1). During each annual Markov cycle the model cohort was distributed across the health states, with these transitions governed by a series of transition matrices (probabilities). Health states were defined in terms of an ‘index’ lesion, that is the greatest malignant potential of the adenoma(s) present, or most advanced cancer present. Individuals with adenomas were classified as low-risk (<10 mm) or higher-risk (⩾10 mm), with the latter category broadly corresponding to the combination of intermediate- and high-risk described by Atkin and Saunders, 2002. Intermediate- and high-risk were not modelled separately owing to limitations in the evidence relating to progression rates through low-intermediate-high risk (Tappenden *et al*, 2004). Discrete cancer states were modelled individually according to AJCC staging. Adenomatous polyps and cancers located in the distal and proximal colon were considered separately to account for the reach of FSIG, with some correlation implicitly modelled by assuming 70% arose in the distal, and 30% in the proximal, colon. Fourteen percent of cancers (based on Munkholm, 2003; Makinen, 2007) were assumed to develop without a prior adenoma (i.e. in individuals with inflammatory bowel disease, or flat or serrated adenomas) and modelled as direct progression from normal epithelium to stage I cancer.

The screening intervention model was superimposed upon the natural history model. The characteristics of the screening (gFOBT, FIT, and FSIG) and diagnostic (colonography, CT colonography) tests were defined in terms of true sensitivity and specificity. The impact of the screening and diagnostic tests, and clinical management of adenomas and cancers, was modelled by redistributing the cohort across the health states at the point of screening or surveillance. Individuals in whom adenomas were detected were assumed to undergo polypectomy and enter surveillance, as described above. Individuals in whom cancer was detected entered a stage-specific clinical management state. Individuals in whom neither cancer nor adenoma was detected were re-invited for screening in the next round, if applicable. Owing to a lack of data, the model assumed that performance characteristics of gFOBT and reflex FIT were independent, and that everyone who had a positive gFOBT completed a reflex FIT.

The mortality model allowed for deaths because of colorectal cancer, endoscopic bowel perforation, or other causes. The probability of dying from other causes was based on Irish life tables and modelled as an age-dependent probability during each Markov cycle. The risk of death from endoscopic perforation was applied during screening (FSIG only), diagnostic investigation, and adenoma surveillance. The probability of dying from colorectal cancer was assumed to be higher for more advanced disease.

The cohort entered the simulation at age 30, at which point it was assumed that prevalence of pre-clinical cancers and adenomas was zero, which is likely to be reasonable for cancers that arise in individuals without specific genetic syndromes (‘sporadic’ cancers). Thus, the prevalence of disease accumulated over the pre-screening period (30–54 or 30–59). The simulation ended at age 100, by which time almost all members were absorbed into the ‘death’ health state.

### Model parameters and calibration

Model parameters were determined from comprehensive literature reviews (published papers supplemented by data from ongoing population-based screening programmes, pilot programmes, and RCTs) and expert opinion if no relevant data was available. For each parameter, we identified a base-case value and range and distribution for use in sensitivity analyses (Table 1).

Estimates of screening uptake, and colonoscopy compliance, were based on the UK pilot programmes and FSIG trial (UK Flexible Sigmoidoscopy Screening Trial Investigators, 2002; Weller *et al*, 2006; Information Services Division, 2008) with various other studies informing the range for sensitivity analyses. Sensitivity and specificity of gFOBT and FIT were derived from pooled analysis of information from diagnostic cohort studies (i.e. the diagnosis had not been determined prior to recruitment, and all participants underwent the index test and reference standard test), which included ‘screening populations’ and, for gFOBT, which used Hemoccult (Beckman Coulter, Inc., Brea, CA, USA) or Hemoccult II (Allison *et al*, 1990, 1996, 2007; Castiglione *et al*, 1991; Foley *et al*, 1992; Itoh *et al*, 1996; Brevinge *et al*, 1997; Chen *et al*, 1997; Nakama *et al*, 2000, 2001; Lieberman and Weiss, 2001; Cheng *et al*, 2002; Niv *et al*, 2002; Gondal *et al*, 2003; Liu *et al*, 2003; Sung *et al*, 2003; Collins *et al*, 2005; Morikawa *et al*, 2005, 2007; Nakazato *et al*, 2006). Three studies were combined to estimate sensitivity of FSIG for intermediate/high-risk adenomas (Rozen *et al*, 1987, Lieberman and Weiss, 2001; Sung *et al*, 2003). As studies included few (if any) low-risk adenomas or cancers, sensitivity estimates for these parameters were based on expert clinical opinion, assuming the former would be lower than for intermediate/high-risk lesions and the latter higher. Specificity was also based on expert opinion. Colonoscopy sensitivity was based on ‘miss rates’ from studies of individuals who underwent tandem colonoscopies (van Rijn *et al*, 2006; Bressler *et al*, 2007), augmented by expert opinion for specificity. CT colonoscopy performance characteristics were from expert opinion informed by reviews and large-scale studies (Cotton *et al*, 2004; Halligan *et al*, 2005; Mulhall *et al*, 2005; Johnson *et al*, 2008).

A healthcare payer perspective was adopted, in this case the Health Service Executive (HSE). Direct costs, in €2008 values, associated with screening and cancer management were included. Costs of gFOBT and FIT kits and associated processing were estimated following discussion with the National Cancer Screening Service, test suppliers, and laboratory staff, and using Department of Health and Children salary scales. The cost of FSIG was estimated from a UK audit (Whynes *et al*, 2003; converted to Euros and inflated using the consumer price index for health) and Irish private health insurer fee schedules. Diagnostic-related group (DRG) costs (HSE Casemix Unit, 2008) were the source for colonoscopy costs. The cost of CT colonography was estimated from expert opinion informed by the fee paid by the HSE for a patient undergoing the procedure in a private facility. Estimation of stage-specific costs of treating (a) screen-detected and (b) symptomatic colorectal cancers is described in detail elsewhere (Tilson *et al*, 2011).

The probability of perforation with FSIG was derived from the UK FSIG Trial (UK Flexible Sigmoidoscopy Screening Trial Investigators, 2002). For colonoscopy, audit data was used to derive estimates for perforation with and without polypectomy (Dafnis *et al*, 2001). The probability of death following perforation came from Gatto *et al* (2003). The UK FSIG Trial informed estimates of episodes of major bleeding following FSIG and colonoscopy (UK Flexible Sigmoidoscopy Screening Trial Investigators, 2002). The costs of treating a bowel perforation and managing a major bleed (which was assumed to result in hospital admission) were estimated from DRG costs (HSE Casemix Unit, 2008).

Health outcomes were measured as quality-adjusted life years (QALYs). Utility for cancer-free individuals was obtained from Fryback and Lawrence (1997) and colorectal cancer stage-specific utility estimates from Ramsey *et al* (2000).

Some model parameters, including the natural history transition probabilities, could not be empirically observed and were obtained by calibration. The approach is described in detail elsewhere (Whyte *et al*, 2011). Briefly, the model was fitted to data on colorectal cancer incidence (by stage) and mortality in Ireland (from the National Cancer Registry and death registrations), and the likely prevalence of adenomas and undiagnosed cancers (estimated from Alexander and Weller, 2003 and Pendergrass *et al*, 2008). Parameters were estimated using Markov Chain Monte Carlo (MCMC) methods and the Metropolis-Hastings algorithm, using a normal likelihood function for observations about mortality, incidence, and prevalence and non-informative Beta(1,1) priors. The model was run using three independent chains with a burn-in of 2000 iterations for each. The set of transition probabilities with the highest likelihood was used in the base-case analysis (Supplementary Table S1). Supplementary Figures 1 and 2 compare actual and model-predicted colorectal cancer incidence and mortality.

### Analysis

Costs and health outcomes associated with spending time in each health state were aggregated over the time horizon to estimate the total cost and health gain associated with each screening option.

In the base-case analysis, costs and health outcomes were discounted at 4% per annum (as recommended for evaluations of health technologies in Ireland; http://www.hiqa.ie) starting at age 55. The marginal cost-effectiveness of each screening scenario compared with the *status quo* (i.e. a policy of no screening) was assessed using incremental cost-effectiveness ratios (ICERs). Scenarios that were not dominated were compared. Although there is no formal cost-effectiveness threshold in Ireland, the HSE have reimbursed most interventions with an ICER <€45 000 per QALY gained.

Selected model parameters were varied in one-way sensitivity analyses. Key parameters varied included those where there is debate (e.g. discount rate; Claxton *et al*, 2011) and/or particular uncertainty (e.g. screening uptake). Probabilistic sensitivity analysis (PSA) was undertaken using Monte Carlo simulation to sample simultaneously from all uncertain model parameters (Table 1). This joint uncertainty was propagated through the model over 1200 iterations (which was sufficient for convergence) to estimate the probability that each screening option was optimal. Natural history parameters were sampled from the parameter sets obtained through calibration, incorporating correlation between these parameters. Most other parameters were treated as independent, but a few which were considered inter-dependent (e.g. test sensitivities for adenomas and cancers) were assigned perfectly correlated distributions.

## Results

### Base-case analysis: core-screening scenarios

No screening was the least expensive option. In the base-case analysis, once-only FSIG at age 60 was expected to be associated with the smallest marginal cost over the lifetime of the cohort compared with no screening (€3.43 per person); this was followed by biennial gFOBT at age 55–74 (€33.63 per person), and biennial FIT at age 55–74 (€40.17 per person; Table 2). The cost of screening (including test kits/examinations, diagnostic procedures, perforations and bleeds, and adenoma surveillance) was similar for gFOBT and FSIG (€56 and €61 per person, respectively), and more than three times higher for FIT (€222 per person).

Compared with no screening, over the lifetime of the cohort all three screening scenarios were associated with a gain in QALYs, which was greatest for FIT (Table 2). All three scenarios appeared to have favourable cost-effectiveness profiles when compared marginally against no screening (i.e. the ICER was significantly lower than the notional cost-effectiveness threshold of €45 000 per QALY). The lowest ICER *vs* no screening was for once only FSIG at age 60 (€589 per QALY gained), followed by FIT at age 55–74 (€1696 per QALY gained) and gFOBT at age 55–74 (€4428 per QALY gained). gFOBT was eliminated by extended dominance: it was more costly than FSIG and less effective than FIT. The ICER for FIT at age 55–74 *vs* once-only FSIG at age 60 was €2058 per QALY, which would be considered favourable.

Over the lifetime of the cohort, compared with no screening, all three scenarios would result in a modest fall in colorectal cancer incidence and a larger fall in colorectal cancer mortality (Table 3). These decreases were expected to be greatest for FIT-based screening (15% fall in incidence, 36% fall in mortality). Faecal immunochemical testing would also result in the largest percentage of screen-detected cases (30%), and smallest percentage of symptomatic cases (68%), in the population (Table 3). All three screening scenarios have the potential to change the stage distribution of cancers in the population, such that a greater proportion would be diagnosed at an early stage. With no screening, the model predicted that 12% of cancers would be stage I at diagnosis, 25% stage II, 35% stage III, and 29% stage IV. With FIT-based screening, 79% of screen-detected and 42% of symptomatic cancers were predicted to be stage I or II; the comparable figures for gFOBT-based screening were 73 and 39%, and for FSIG screening were 71 and 37%.

The FSIG-based screening would result in a higher lifetime rate of endoscopy procedures than screening based on faecal testing, but most of these would be FSIG screening examinations (Table 4). The rate of colonoscopies over the lifetime of the cohort would be ten times higher (34 632 *vs* 3386 per 100 000), and that of polypectomies eight times higher (9486 *vs* 1125 per 100 000), for FIT-based screening than for screening based on gFOBT. A consequence of this would be a higher rate of complications with FIT-based screening than the other options (Table 4).

### Sensitivity analyses

When costs and benefits were not discounted, all three screening scenarios appeared more cost-effective. The ICERs compared with no screening were: FIT at age 55–74, -€1399 per QALY gained; gFOBT at age 55–74, €410 per QALY gained; and FSIG at age 60, −€2012 per QALY gained.

**Figure 2:**
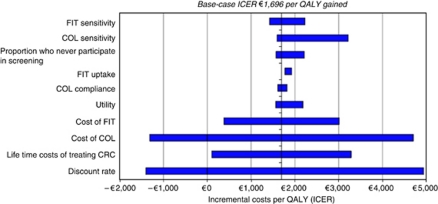
Incremental costs per QALY compared with no screening, when selected parameters were varied independently, for biennial FIT at 55–74 years. Abbreviations: COL=colonoscopy; CRC=colorectal cancer; FIT=faecal immunochemical test.

Figure 2 shows the results of the one-way sensitivity analysis for FIT. In addition to discount rate, the most influential parameters were costs of screening tests and costs of managing colorectal cancer. However, even when these were varied, the ICER for FIT *vs* no screening remained very much below the notional cost-effectiveness threshold, and in some instances became cost saving (ICER<0). Varying screening uptake had very little impact on cost-effectiveness. The results of the one-way sensitivity analyses for FSIG and gFOBT were similar to those for FIT (i.e., the same parameters had the greatest impact, and in all instances, the ICERs for screening *vs* no screening remained very much below the notional cost-effectiveness threshold (data not shown)).

**Figure 3:**
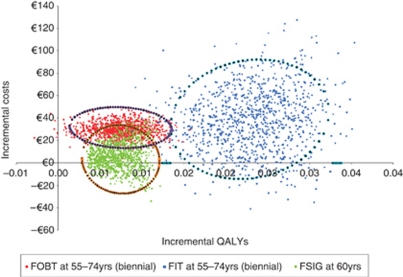
PSA: incremental costs and incremental QALYs with 95% confidence ellipses, for core screening scenarios compared with no screening. Abbreviations: FIT=faecal immunochemical test; FSIG=flexible sigmoidoscopy; gFOBT=guaiac-based faecal occult blood test.

Figure 3 shows the results of the PSA. In only a handful of simulations, all pertaining to gFOBT, screening was expected to result in a loss of QALYs compared with no screening. Uncertainty was greatest for FIT, but the outlying simulations remained well below the notional cost-effectiveness threshold. In almost every instance, the incremental costs of screening with gFOBT exceeded those for FSIG, whereas the incremental QALYs for FIT exceeded those for the other two scenarios. Thus, the findings from the base-case analysis were confirmed i.e., (1) all three scenarios were almost always likely to be considered highly cost-effective compared with no screening; and (2) screening with FIT was likely to result in the greatest health gain and would, therefore, be considered the optimal strategy.

### Age-variant scenarios

The cost-effectiveness results of the base-case analyses for the five age-variant scenarios are shown in Table 2. All variant scenarios had favourable cost-effectiveness profiles compared with no screening. For faecal testing, the ICER *vs* no screening was lower for screening restricted to the younger (55–64 years), compared with the full (55–74) age group. The FIT-based scenarios were more cost-effective, *vs* no screening, than the gFOBT scenarios. For FSIG, offering screening at 55 years was less cost-effective than at age 60. The only strategies not eliminated by extended dominance were, in order of incremental QALYs gained: FSIG at age 60, FIT at 55–64 years, and FIT at 55–74 years. The ICER for FIT at 55–64 years *vs* FSIG at 60 years was €1436 per QALY gained. The ICER for FIT at 55–74 years compared with FIT at 55–64 years was €3221 per QALY gained, indicating that FIT in the full age group (55–74 years) remained the most cost-effective strategy. This was confirmed in PSA (Data not shown). The cost-effectiveness acceptability curve (Data not shown) showed that if decision makers were willing to pay a maximum of around €1000 per additional QALY, the most cost-effective strategy would be expected to be FSIG at age 60. At a threshold of ⩾€4000 per additional QALY, the optimal option would be biennial FIT at 55–74 years (at €4000, *P*=0.693 that this is the most cost-effective strategy; at €6000, *P*=0.907; at €8000, *P*=0.961, at €14 000, *P*=0.990).

## Discussion

The key issues in deciding whether to introduce a new screening programme include whether: (1) screening represents a cost-effective intervention (i.e., the health gains are likely to be significant compared with the costs involved); (2) uptake is likely to be sufficiently high for screening to be effective; and (3) implementation is feasible (i.e., sufficient health service resources are available to diagnose, treat and follow-up those found to have adenomas and cancer).

### Cost-effectiveness

This analysis clearly shows that a population-based colorectal cancer screening programme in Ireland – using gFOBT, FIT, or FSIG – would be likely to be considered highly cost-effective compared with no screening. This is generally consistent with the conclusions from most previous cost-effectiveness evaluations (HIQA, 2009; and references therein; Berchi *et al*, 2010; Lejeune *et al*, 2010; Telford *et al*, 2010; Hassan *et al*, 2011; van Rossum *et al*, 2011). Estimates from individuals studies are not entirely comparable, not least because the screening scenarios evaluated, and the outcomes and comparators, differ. Nonetheless, the ICERs in the current study were generally somewhat lower than to those reported elsewhere. For example, in a French study, the ICER for biennial FIT was €8589 per life year saved (Hassan *et al*, 2011) compared with €1696 per QALY gained in this analysis. These differences are probably because of the rising costs of colorectal cancer treatment (Schrag, 2004). We included costs of combination chemotherapies and monoclonal antibodies, which are expensive, but now part of standard care, and our estimated treatment costs were higher than those reported in older studies from other European countries (Tilson *et al*, 2011). One consequence of the rising treatment costs is that screening could be considered desirable not only in terms of reducing colorectal cancer incidence and mortality, but also as a means to control treatment costs (Lansdorp-Vogelaar *et al*, 2009).

In this analysis, biennial FIT at 55–74 years dominated biennial gFOBT for the same age range, findings similar to van Rossum *et al* (2011), whose analysis was based on empirical RCT data. Importantly, in light of recent evidence that FSIG is effective in reducing cancer incidence and mortality (Atkin *et al*, 2010; Segnan *et al*, 2011), we found that once-only FSIG had the lowest ICER compared with no screening. However, FIT was associated with much larger health gains and, on this basis, would be considered the optimal option.

### Uptake

Previous cost-effectiveness analyses of colorectal cancer screening have been criticised for including overly optimistic estimates of screening uptake and compliance with diagnostic investigations, leading to uncertainty in the true cost-effectiveness of screening (van Rossum *et al*, 2011). The estimates of participation in the current study were chosen because they were considered to be plausible (53% for faecal tests based on the UK pilot programmes and 39% for FSIG based on the UK trial) (UK Flexible Sigmoidoscopy Screening Trial Investigators, 2002; Weller *et al*, 2006; Information Services Division, 2008). In addition, we assumed that uptake would be the same for FIT and gFOBT because, although some studies have suggested that uptake could be higher with FIT than gFOBT (Cole and Young, 2001; Deutekom *et al*, 2010; Hol *et al*, 2010), this is not a universal finding (Levi *et al*, 2011). We varied uptake in sensitivity analyses and the relative cost-effectiveness of screening changed little; this is because, as well as reducing costs, lower uptake also reduces effectiveness. For example, with 70% FIT uptake the ICER was €1771 per QALY gained compared with €1696 in the base-case. Thus, had we assumed that FIT uptake exceeded that for gFOBT, our main conclusions would have been unchanged.

Although they do not impact greatly on cost-effectiveness, high uptake rates are essential if screening is to be effective in reducing mortality in the population. Whether the participation levels from the analysis are achievable in Ireland is unclear. On one hand, the base-case estimates were higher than uptake in some Europe programmes (Peris *et al*, 2007; Kis, 2010; Zorzi *et al*, 2010). On the other hand, 51% of 9993 individuals aged 50–74 years resident in Dublin and invited to complete a FIT did so (McNamara *et al*, 2011), suggesting the FIT uptake level considered in our analysis is realistic and attainable.

Screening programmes also require high levels of compliance with diagnostic tests and surveillance following polypectomy. Our base-case estimate for colonoscopy compliance was derived primarily from the UK screening programmes and very close to the level reported in the Dublin study (McNamara *et al*, 2011). However, most data relates to diagnostic colonoscopy and compliance with surveillance remains uncertain. Although (as our analysis shows) low compliance with surveillance would have a relatively modest influence on cost-effectiveness, it would adversely impact on programme effectiveness.

### Feasibility

Cost-effectiveness should not be considered in isolation to issues relating to service delivery, and although FIT in age 55–74 years was considered the optimal option, such a programme would require substantial more resources for colonoscopy (and CT colonography and other diagnostic procedures) than one based on gFOBT or FSIG. An option appraisal in England, based on a similar economic model to the current analysis, recognised the importance of endoscopy resource and capacity issues, and suggested that gFOBT-based, although less cost-effective, was probably more feasible than a programme based on FSIG (FIT was outwith the scope of the analysis; Tappenden *et al*, 2004). In the UK pilot programmes, which are likely to be much less resource intensive than a programme based on FIT, achieving sufficient colonoscopy capacity has been a major challenge, and has underpinned the age and area-based rollout. Hence, any programme that adopts primary screening by FIT will have to consider very carefully how to deliver sufficient capacity for diagnosis and surveillance. One option to address capacity issues would be to consider restricting screening to a narrower age range. We found that screening at 55–64 years had a lower ICER than screening over 55–74 years, although the gain in QALYs was not as large. Age-restricted FIT-based screening could be an attractive strategy therefore, not only for Ireland but also elsewhere. Of course, this is only one of a range of possible implementation options; others might include rollout area by area or across age groups until the full country/age range is incorporated or setting a high FIT cut-off level for colonoscopy referral. In Ireland, on the basis of the cost-effectiveness results reported here, a decision was taken to implement a FIT-based colorectal cancer screening programme from 2012; in the short-term, a restricted age range (60–69 years) will be invited to participate but the stated intention is to eventually include the entire 55–74 years age group (National Cancer Screening Service, 2011).

A related issue concerns adverse effects of endoscopy among screening participants. Because of the high rate of colonoscopies and polypectomies, screening based on FIT would be associated with a much higher lifetime risk of major abdominal bleeding, bowel perforation, and death than screening with gFOBT or FSIG. However, even with FIT-based screening the absolute risk to an individual of experiencing one of these complications is low, and in ongoing programmes major complications of colonoscopy are rare (Regula *et al*, 2006; Information Services Division, 2008)

### FIT *vs* gFOBT

The FIT is increasingly being adopted as a primary test in screening programmes. As this strategy was more cost-effective than screening by gFOBT with reflex FIT (currently implemented in Scotland) this raises the question of whether the UK screening programmes should use primary FIT testing. Dealing with advances in the evidence base is always a challenge for existing screening programmes. The efficacy of gFOBT in reducing colorectal cancer mortality is established (Hewitson *et al*, 2007) but FIT may (or may not) be more sensitive and more cost-effective. We derived our estimates of the performance characteristics of gFOBT from diagnostic cohort studies of Hemoccult and Hemoccult II. Other, newer, gFOBTs may have a higher sensitivity (van Dam *et al*, 2010) and the potential to be more effective. We repeated our analysis using higher estimates of gFOBT sensitivity obtained from a study using Hemoccult Sensa (adenomas, 20%, cancers, 64% Allison *et al* 2007). The ICER compared with no screening was €1701 per QALY gained – very close to that for screening using FIT (€1696 per QALY gained). Therefore, it is entirely possible that a screening programme based on gFOBT could achieve similar health gains to one based on FIT, if a sufficiently sensitive test was used.

### Comparisons with results of RCTs

Our estimate of the mortality reduction achieved with gFOBT-based screening (12%) was slightly lower than RCT results (16% Hewitson *et al*, 2007). Our estimate of the effect of FSIG screening on colorectal cancer incidence (5% reduction) was higher than in one trial (Hoff *et al*, 2009), but lower than in others (Atkin *et al*, 2010; Segnan *et al*, 2011). There are numerous reasons why findings from RCTs and cost-effectiveness analyses might not entirely correspond, including significant differences between the: trial participants and population eligible for screening in Ireland; time horizons; diagnostic and surveillance protocols; and values of key parameters (e.g. test sensitivity, and uptake).

### Strengths and limitations

Unlike most previous natural history models, we assumed that some cancers (14%) would arise without a prior adenoma. However, the frequency and malignant potential of hyperplastic and flat polyps in European populations is uncertain. Thus, if >86% of cancers develop though the adenoma-carcinoma sequence, our model is likely to have underestimated screening effectiveness, with the extent of underestimation differing for faecal and endoscopic tests.

Important questions remain about the efficacy and effectiveness of the screening and diagnostic tests considered here. Even for gFOBT, which has been extensively investigated, there remains a lack of certainty about true performance characteristics, particularly for the newer versions of the test. The estimation of sensitivity and specificity of FIT in population-based screening is hampered by the fact that numerous tests are available with heterogeneous performance characteristics, and various approaches have been taken to estimate sensitivity, a criticism that has been made previously (Burch *et al*, 2007). In addition, although quantitative FITs, theoretically, allow the level to define a ‘positive’ result to be set for individual populations and in accordance with local circumstances (e.g. to suit available colonoscopy capacity; Fraser *et al*, 2008) the absence of high-quality data available at the time we parameterised the models meant that we did not estimate cost-effectiveness of different cut-offs. In effect, we assessed cost-effectiveness at 100 ng ml^−1^ as this was the cut-off in the key quantitative studies which informed the parameter estimates. A recent analysis suggests that FIT may be even more cost-effective at a cut-off of 50 ng ml^−1^ than at 100 ng ml^−1^ (Wilschut *et al*, 2011). In addition, some recent studies suggest that sensitivity of FIT might exceed the values used in the analysis, particularly at low cut-offs (see, for example, Omata *et al*, 2011; Rozen *et al*, 2011). The impact of higher sensitivity on cost-effectiveness is twofold – it increases the number of lesions detected and increases costs. Therefore, as test sensitivity is not a major driver of cost-effectiveness, if we were to repeat our analysis based on these newer studies, it is likely that our overall conclusions would be unchanged.

As regards FSIG, there are few studies of sensitivity and specificity, and the gold standard (colonoscopy) misses lesions (van Rijn *et al*, 2006; Bressler *et al*, 2007). This means that such studies will tend to overestimate FSIG performance. Moreover, colonoscopy performance characteristics are uncertain because it is usually considered the gold standard for endoscopic evaluation. In light of this uncertainty, it was reassuring that our overall conclusions were unchanged after extensive sensitivity analyses. However, it should be noted that the ranges for the parameter values used in the sensitivity analyses were sometimes informed by expert opinion because robust data was lacking.

We chose to evaluate a strategy that combined gFOBT with reflex FIT, instead of the more conventional approach of reflex gFOBT. This was because second-line FIT has been shown to limit the number of colposcopy referrals (Fraser *et al*, 2006, 2007). However, we accept that the strategy is not widely used. Our analyses were based on QALYs and the model incorporated quality-of-life decrements associated with colorectal cancer. It is entirely possible that screening might also impact adversely on quality-of-life (e.g., in those with a positive screening test or those undergoing annual surveillance), but we were unable to identify any robust utility data for these health states. Thus, our study (and other similar studies) may somewhat overestimate benefits of screening. In common with similar analyses, we did not include costs of setting up programme infrastructure and some costs associated with ongoing programme administration and delivery. Many of these depend on the business model adopted. Because of the limited evidence base, costs incurred by screening participants (such as travel costs) and societal costs (such as lost productivity) were not included. This would tend to mean that our cost estimates are conservative. It is important, however, to acknowledge that all of these costs exist and are likely to vary for different screening modalities.

A major area of uncertainty in this and other similar models relates to the true underlying population prevalence of adenomas. We estimated prevalence on the basis of data from a recent, large, well-conducted, autopsy study and the first round of the pilot screening programmes in Scotland and England (Alexander and Weller, 2003; Pendergrass *et al*, 2008). Our estimates of prevalence were lower than those from older autopsy series, which other analyses have used. The prevalence estimates from these older studies vary greatly (Tappenden *et al*, 2004) and they have been criticised for being small, providing little information on the source population, and not always clearly distinguishing between different types of polyp (Jass *et al*, 1992). It is impossible to be sure which of the available sources is closer to the true prevalence of adenomas.

Finally, although cost-effectiveness analysis is a valuable tool for comparing costs and benefits of alternative screening options, it may not fully address ‘real world’ issues around programme implementation and delivery. For example, such analyses do not provide annual estimates of health service resource requirements for the actual population eligible for screening. Alternative approaches to running economic models can be used to obtain this type of information (see, for example, Health Information and Quality Authority, 2009).

## Conclusions

This analysis suggests that a screening programme based on biennial screening at 55–74 years with FIT would be preferable to one based on biennial gFOBT (with reflex FIT) at 55–74 years or once-only FSIG at 60 years. Although a programme based on FIT is expected to result in the greatest health improvement, it would require more colonoscopy resources and result in more individuals suffering adverse effects. The major challenges for policy makers are, therefore, balancing the benefits and harms of screening while ensuring sufficient capacity for follow-up of screen-detected adenomas and cancers.

## Figures and Tables

**Table 1:** Parameter estimates, with base-case values, ranges and distributions

**Model parameter**	**Base-case**	**Range for SA**	**Distribution for PSA^a^**	**Source**
*Performance of screening tests*				
gFOBT sensitivity for adenomas	11%	10–12%	Beta (11.40, 92.10)	Allison *et al*, 1990; Castiglione *et al*, 1991; Foley *et al*, 1992; Allison *et al*, 1996; Brevinge *et al*, 1997; Lieberman and Weiss, 2001; Niv *et al*, 2002; Sung *et al*, 2003 Collins *et al*, 2005
gFOBT sensitivity for CRC	36%	31–42%	Beta (105.00, 186.60)	
gFOBT specificity for adenomas and CRC	97%	96–98%	Beta (1083.40, 33.50)	
FIT sensitivity for adenomas	21%	19–22%	Beta (594.62, 2236.92)	Allison *et al*, 1996; Itoh *et al*, 1996; Chen *et al*, 1997; Nakama *et al*, 2000; Nakama *et al*, 2001; Cheng *et al*, 2002; Gondal *et al*, 2003; Liu *et al*, 2003; Morikawa *et al*, 2005; Nakazato *et al*, 2006; Allison *et al*, 2007; Morikawa *et al*, 2007
FIT sensitivity for CRC	71%	67–75%	Beta (35.29, 143.08)	
FIT specificity for adenomas and CRC	95%	94–96%	Beta (1732.57, 91.19)	
FSIG sensitivity for low-risk distal adenomas	65%	60–70%	Beta (235.00, 126.54)	Expert opinion informed by Rozen *et al*, 1987; Lieberman and Weiss, 2001; Sung *et al*, 2003
FSIG sensitivity for intermediate/high-risk distal adenomas	74%	68–78%	Beta (180.00, 63.24)	
FSIG sensitivity for distal CRC	90%	85–95%	Beta (90.00, 10.00)	
FSIG specificity for distal adenomas and CRC	92%	90–95%	Beta (250.00, 21.74)	
				
*Uptake and compliance with screening and diagnostic tests*				
gFOBT uptake	53%	32–70%	Uniform (32%, 70%)	Weller *et al*, 2006; Information Services Division, 2008
FIT uptake	53%	32–70%	Uniform (32%, 70%)	
FSIG uptake	39%	24–67%	Uniform (24%, 67%)	UK Flexible Sigmoidoscopy Screening Trial Investigators, 2002
% of individuals who never accept an offer of screening^b^	13%	0–41%	—	Weller *et al*, 2006
COL compliance (diagnostic test)	86%	81–90%	Uniform (81%, 90%)	Weller *et al*, 2006; Information Services Division, 2008
				
*Performance of diagnostic tests and related parameters*				
COL sensitivity for low-risk adenomas	77%	73–80%	Beta (350.00, 104.55)	van Rijn *et al*, 2006 Bressler *et al*, 2007
COL sensitivity for intermediate/high-risk adenomas	98%	93–99%	Uniform (93%, 99%)	
COL sensitivity for CRC	98%	95%–99%	Uniform (95, 99%)	
COL specificity for adenomas and CRC	97%	96–98%	Beta (970.00, 30.00)	Expert opinion
CTC sensitivity for low-risk adenomas	53%	45–60%	Beta (80.00, 70.94)	Expert opinion, informed by Cotton *et al*, 2004; Halligan *et al*, 2005; Mulhall *et al*, 2005; Johnson *et al*, 2008
CTC sensitivity for intermediate/high-risk adenomas	85%	48–100%	Beta (4.50, 0.79)	
CTC sensitivity for CRC	85%	75–95%	Beta (50.00, 8.82)	
CTC specificity for adenomas and CRC	86%	80–90%	Beta (140.00, 22.79)	
Average number of adenomas removed per person	1.9	—	—	Winawer *et al*, 1993
				
*Surveillance of screening-detected adenomas*				
% of those in with intermediate/high-risk adenomas removed in whom the adenoma was high-risk	29%	—	—	Alexander and Weller, 2003; Weller *et al*, 2006
COL compliance (surveillance)	86%	81–90%	Uniform (81%, 90%)	Assumption based on Weller *et al*, 2006; Information Services Division, 2008
				
*Harms of screening*				
FSIG probability of perforation (with or without polypectomy)	0.002%	0–0.051%	Uniform (0%, 0.051%)	UK Flexible Sigmoidoscopy Screening Trial Investigators, 2002
FSIG probability of death following perforation	6.452%	0–9.070%	Uniform (0%, 9.070%)	Gatto *et al* (2003)
Probability of (major) bleeding following FSIG	0.029%	0.002–0.054%	Uniform (0.002%, 0.054%)	UK Flexible Sigmoidoscopy Screening Trial Investigators, 2002
COL probability of perforation (with polypectomy)	0.216%	0.168–0.298%	Uniform (0.168%, 0.298%)	Dafnis *et al*, 2001
COL probability of perforation (without polypectomy)	0.107%	0.010–0.249%	Uniform (0.010%, 0.249%)	
COL probability of death following perforation	5.195%	0–9.070%	Uniform (0%, 9.070%)	Gatto *et al* (2003)
Probability of (major) bleeding following COL	0.379%	0.065–0.412%	Uniform (0.065%, 0.412%)	UK Flexible Sigmoidoscopy Screening Trial Investigators, 2002
				
*Health-related QoL*				
Utility: cancer free	0.94	—	—	Fryback and Lawrence (1997)
Utility: stage I, II, III, IV cancer	0.80	0.43–0.94	0.94^*^Beta (3.92, 0.69)	Ramsey *et al* (2000)
				
*Costs*				
gFOBT kit^c^	€1.70	€1.36–€2.04	Uniform (€1.36, €2.04)	Estimated by authors
gFOBT processing/analysis^d^	€7.81	€6.25–€9.37	Uniform (€6.25, €9.37)	
FIT kit^c^	€3.75	€3–€4.50	Uniform (€3, €4.50)	
FIT processing/analysis^d^	€11.60	€9.28–€13.92	Uniform (€9.28, €13.92)	
Cost of FSIG (with/without polypectomy)	€150	€120–€180	Uniform (€120, €180)	Whynes *et al*, 2003; VHI Healthcare
Cost of COL	€650	€520–€780	Uniform (€520, €780)	HSE Casemix Unit, 2008
Cost of CTC	€550	€440–€660	Uniform (€440, €660)	Expert opinion
Cost of treating bowel perforation	€10 200	€8160–€12 240	Uniform (€8160, €12 240)	HSE Casemix Unit, 2008
Cost of admittance for bleeding	€3079	€2463–€3695	Uniform (€2463, €3695)	
Pathology cost for adenoma	€65	€52–€78	Uniform (€52, €78)	Tappenden *et al*, 2004
Pathology cost for cancer	€530	€424–€636	Uniform (€424, €636)	
Lifetime cost stage I CRC-symptomatic	€23 688	€18 950–€28 425	Uniform (€18 950, €28 425)	Tilson *et al*, 2011
Lifetime cost stage II CRC-symptomatic	€37 180	€29 744–€44 616	Uniform (€29 744, €44 616)	
Lifetime cost stage III CRC-symptomatic	€48 835	€39 068–€58 602	Uniform (€39 068, €58 602)	
Lifetime cost stage IV CRC-symptomatic	€36 602	€29 281–€43 922	Uniform (€29 281, €43 922)	
Lifetime cost stage I CRC-screen-detected	€22 885	€18 308–€27 462	Uniform (€18 308, €27 462)	
Lifetime cost stage II CRC-screen-detected	€36 377	€29 102–€43 652	Uniform (€29 102, €43 652)	
Lifetime cost stage III CRC-screen-detected	€48 032	€38 426–€57 638	Uniform (€38 426, €57 638)	
Lifetime cost stage IV CRC-screen-detected	€35 799	€28 639–€42 959	Uniform (€28 639, €42 959)	
				
*Discount rate*				
Discount rate for costs and benefits	4%	0–6%	—	Recommended for Ireland

Abbreviations: COL=colonoscopy; CRC=colorectal cancer; CTC=CT colonography; FIT=faecal immunochemical test; FSIG=flexible sigmoidoscopy; gFOBT=guaiac-based faecal occult blood test; PSA=probabilistic sensitivity analysis; SA=sensitivity analysis; low-risk adenoma(s), <10 mm; intermediate/high-risk adenoma(s), ⩾10 mm.

a if no distribution given, parameter was not varied in the PSA.

b relevant to gFOBT and FIT scenarios only.

c cost per kit dispatched (i.e., cost per individual invited to participate in screening).

d cost per kit completed and returned (i.e., cost per screening participant).

**Table 2:**
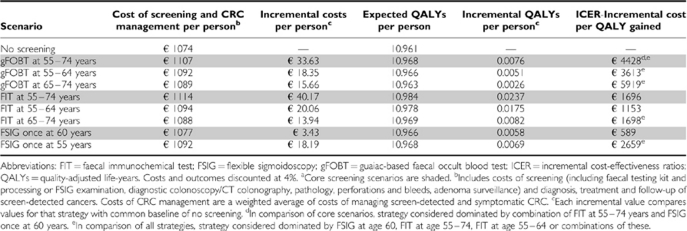
ICER, based on QALYs per person, for core^a^ and age-variant screening scenarios

**Table 3:** Lifetime rates^a^ of colorectal cancer incidence and mortality per 100 000 population, percentage of cases which would be detected by screening, surveillance and symptomatically, and percentage reductions in incidence and mortality compared with no screening, for core screening scenarios

	**Incidence**								
	**Screen detected CRC**		**Surveillance-detected CRC^b^**		**Symptomatic CRC**			**Mortality**	
**Scenario**	**Rate**	**% of cases**	**Rate**	**% of cases**	**Rate**	**% of cases**	**% reduction in CRC incidence^c^**	**CRC mortality rate**	**% reduction in CRC mortality^c^**
No screening	0	—	0	—	5158	100	—	2287	—
gFOBT at 55–74 years	695	13.6	11	0.2	4401	86.2	1.0	2016	11.8
FIT at 55–74 years	1313	29.8	78	1.8	3010	68.4	14.7	1465	36.0
FSIG once at 60 years	138	2.8	25	0.5	4742	96.7	4.9	2116	7.5

Abbreviations: CRC=colorectal cancer; FIT=faecal immunochemical test; FSIG=flexible sigmoidoscopy; gFOBT=guaiac-based faecal occult blood test.

a Over the entire lifetime of the cohort, therefore, for gFOBT and FIT includes 10 screening rounds.

b CRC detected at surveillance among those with intermediate/high-risk adenomas found at screening.

c Each incremental value compares values for that strategy with common baseline of no screening.

**Table 4:** Lifetime rates^a^ per 100 000 population of screening-related endoscopic procedures^b^, and associated complications^c^, for the core screening scenarios

	**Endoscopic procedures**			**Complications**		
**Scenario**	**Flexible sigmoidoscopy**	**Colonoscopy**	**Polypectomy**	**Major bleeding^d^**	**Bowel perforation**	**Deaths due to perforation**
gFOBT at 55–74 years	—	3386	1215	12	5	0.26
FIT at 55–74 years	—	34 632	9486	132	57	3.00
FSIG once at 60 years	40 177	2543	2487	22	5	0.25

Abbreviations: FIT=faecal immunochemical test; FSIG=flexible sigmoidoscopy; gFOBT=guaiac-based faecal occult blood test.

a Over the entire lifetime of the cohort, therefore, for gFOBT and FIT includes 10 screening rounds.

b Related to screening, diagnosis, or surveillance.

c Complications associated with diagnostic and surveillance colonoscopy and, where relevant, FSIG.

d Major abdominal bleeding, requiring admission or intervention.
