# Supplementary material for: Cost-effectiveness of population-based screening for colorectal cancer: a comparison of guaiac-based faecal occult blood testing, faecal immunochemical testing and flexible sigmoidoscopy
Source: Br J Cancer. 2012 Feb 16;106(5):805–16. doi: 10.1038/bjc.2011.580 (PMC3305953; doi:10.1038/bjc.2011.580)
Supplement: Supplementary Information [file bjc2011580x1.doc]

**Supplementary figure 1. Results of model calibration**

| **(a) Actual^1^ and model predicted stage-specific incidence of colorectal cancer** | **(b) Actual^1^ and model predicted colorectal cancer mortality rates** |
| --- | --- |
|  |  |

^1^ from national cancer registrations and death registrations for Ireland

**Supplementary table 1. Natural history parameters estimated from model calibration process^1^ and used in base-case analysis**

| ***Parameter*** | ***Value*** |
| --- | --- |
| *Adenoma and cancer annual transition probabilities* |  |
| Normal epithelium to low-risk adenoma^2^ | age 30: 0%  age 70: 1.07%  age 100: 1.04% |
| Low-risk adenoma to intermediate/high-risk adenoma | 5.73% |
| Intermediate/high-risk adenoma to stage I cancer | 5.82% |
| Stage I to stage II cancer | 90.47% |
| Stage II to stage III cancer | 72.00% |
| Stage III to stage IV cancer | 63.12% |
| *Symptomatic cancer presentation*: probability of presenting symptomatically with: |  |
| stage I cancer | 23.80% |
| stage II cancer | 32.16% |
| stage III cancer | 48.14% |
| stage IV cancer | 90.41% |
| *Mortality rates*: annual colorectal cancer-specific mortality rate for |  |
| stage I cancer | 0.23% |
| stage II cancer | 0.65% |
| stage III cancer | 4.03% |
| stage IV cancer | 30.49% |

^1^ based on the parameter set with the greatest likelihood

^2^ this is an age dependent variable; probability increases from age 30 to 70, and falls slightly thereafter. These values are examples from the parameter set.
